# Medical Clearance of Older Adults Participating in Preventative Direct Access Physical Therapy

**DOI:** 10.7759/cureus.35784

**Published:** 2023-03-05

**Authors:** Sara K Arena, Christopher M Wilson, Lori Boright, Olivia Webster, Carly Pawlitz, Caitlin Kovary, Emily Esper

**Affiliations:** 1 Physical Therapy, Oakland University, Rochester, USA; 2 Rehabilitation Services, Beaumont Health, Troy, USA

**Keywords:** prevention, physical therapy, cognition screening, cardiovascular screening, exercise

## Abstract

Background

The purpose of this study was to determine if the use of evidence-based cognitive and cardiovascular screening prior to initiating a prevention-focused exercise program that utilizes a physical therapist (PT) direct consumer access referral model is safe.

Methods

A retrospective descriptive analysis of data from a prior randomized controlled trial (RCT) was performed. Two data sets emerged: Group S was screened for study inclusion but not enrolled, and Group E was enrolled and participated in preventative exercise. Participant outcomes of cognitive screenings (Mini-Cog, Trail Making Test-Part B) and cardiovascular screening (American College of Sports Medicine Exercise Pre-participation Health Screening) were extracted. Descriptive statistics were generated for demographic and outcome variables and inferential statistics were analyzed (p < 0.05).

Results

Records from 70 individuals (Group S) and 144 individuals (Group E) were available for analysis. Overall, 18.6% (n = 13) in Group S were not enrolled due to medical instability or potential safety considerations. The need for medical clearance prior to initiating an exercise program was identified and then clearance was obtained for 40% (n = 58) of the participants in Group E. No adverse events related to program participation were reported.

Conclusions

A PT-led program utilizing direct access referrals from senior centers offers a safe option for older adults to participate in individualized preventative exercise programming.

## Introduction

In all 50 U.S. states, individuals are permitted to actively seek out physical therapist (PT) services without a physician referral or prescription. In January 2015, the state of Michigan became the final state to pass legislation to permit direct consumer access to PT services [1]. While there are variations to direct access provisions within each state’s jurisdiction, Michigan allows physical therapy treatment without a physician’s prescription for the purposes of injury prevention and fitness promotion and does not pose time or visit limitations to prevention-focused interventions [2].

In a recent analysis of practice patterns in physical therapy, it was determined that 40-43% of patients receiving physical therapy across practice settings were age 65 or older [3]. According to the 2019 Profile of Older Americans, people aged 65 and older represented 16% of the population, but growth in this population segment is anticipated to grow to 21.6% by 2040 [3]. As the U.S. population continues to age, older adults will comprise the largest percentage of patients/clients across most medical practice settings, including physical therapy [4]. Advancing age increases the presence of disease and the risk of a fall with the potential for subsequent injuries. Therefore, prevention-focused efforts aimed at fall reduction and health promotion are warranted for older adults. Direct consumer access options that target these risks have evidence for their efficacy and should be among the strategies considered to reduce physical and economic burdens [5]. Notably, a study by Demont et al. suggested that PT services provided through direct access and patient self-referral could provide better outcomes in terms of decreasing disability, improving quality of life, and reducing healthcare costs compared to physician-led medical care for patients with musculoskeletal disorders [6]. However, this systematic review was focused on impairment-based direct access PT services and there remains a paucity of evidence for prevention-focused PT direct access care models for older adults.

An important role of PTs is to prescribe and dose therapeutic exercise, which is a skill requiring extensive knowledge of the multitude of risks associated with engagement in exercise and physical activity. Due to a potential increased risk of falls when performing exercise (i.e., standing balance exercises), there are no clear guidelines related to allowing the performance of these activities for people with cognitive impairments. While there is a benefit to physical activity and exercise for most individuals, it is unclear what safety risk may be introduced when providing a home exercise program inclusive of balance challenges to those with cognitive impairment. Additionally, older adults who participate in exercise and have an elevated cardiovascular-related risk or known disease may be at an increased risk to experience medical or safety events during exercise [7]. Therefore, screening of individuals prior to initiating exercise, including prevention-focused exercise, is recommended to assure safe exercise participation [7]. This is particularly important when utilizing direct consumer access referrals to PTs as these professionals may be the first point of contact for an individual seeking preventative-focused skilled healthcare services.

Evidence-based screenings are foundational tools that are useful to guide a PT’s clinical judgment [8]. The direct access referral mechanism warrants a focus on these tools and attention to specific and sensitive screening tools that assure safe initiation of exercise participation. Importantly, tools that examine the cardiovascular and cognitive status and safe readiness to engage in activity should be regularly utilized prior to the initiation of exercise interventions. The American College of Sports Medicine (ACSM) Exercise Pre-participation Health Screening tool is useful to identify individuals who may be at an elevated risk for exercise-related cardiac death and/or myocardial infarction, especially in those with underlying cardiovascular or pulmonary disease [9]. The tool utilizes questions aimed at garnering insight into current symptoms, current engagement in and volume of physical activity, and the person’s medical history. Responses may result in the need for medical clearance by a physician prior to exercise engagement if an elevated risk is identified.

One example of a program that utilized pre-participation cardiovascular and cognitive screening prior to enrolling older adults in a direct access prevention program is the Home-Based Older Persons Upstreaming Prevention Physical Therapy (HOP-UP-PT) program. This article provides a secondary analysis of the screening components of a HOP-UP-PT clinical trial carried out in Michigan. This program utilizes community-based senior center referrals of an older adult directly to a PT who first screens the individual for safe program participation. Once participation is deemed safe, outcomes of a detailed evaluation guide the PT to develop an individualized home-based plan of care which includes physical, environmental, and health-related interventions aimed at optimizing successful aging in place [10,11]. A randomized controlled trial of 144 older adults who participated in the HOP-UP-PT program demonstrated evidence of an eight-fold reduction in falls after participating in the multimodal program which included exercise [12]. The HOP-UP-PT program utilized evidenced-based risk screening tools including a standardized pre-participation telephone and in-person health screening. Specifically, the ACSM Exercise Pre-participation Health Screening, MiniCog, and Trail Making Test Part B (TMT) were among the tools that HOP-UP-PT employed to evaluate safety risk prior to engaging in exercise and technology use [9,13,14]. Details of each of these tools are provided in the methods section of this paper. However, an examination of how and at what volume PTs may be contributing to identifying potentially serious medical issues among older adults receiving prevention-focused programming through a direct access care model requires investigation. Therefore, the purpose of this study was to determine if the use of evidence-based cognitive and cardiovascular screening prior to initiating a prevention-focused exercise program that utilizes a PT direct consumer access referral model is safe.

The contents of this article were previously presented as a meeting abstract at the 2023 American Physical Therapy Association Combined Section Meeting on February 25, 2022.

## Materials and methods

Research design

After securing Oakland University Institutional Review Board approval (#FY2021-414 and #FY2022-11), a retrospective descriptive analysis of data obtained but not analyzed during a prior randomized controlled clinical trial (RCT) was initiated.

Sampling criteria

Two unique datasets were available from the prior RCT for analysis. Inclusion in the RCT required participants to be greater than or equal to 65 years of age, identified by the referral source to be subjectively at risk for functional decline or falls, and to express a willingness to participate in the HOP-UP-PT program. Individuals were excluded if they had been hospitalized or had received physical therapy services within the prior two months or were currently receiving palliative or hospice care. The first dataset was from 70 individuals (Group S) who were screened for potential enrollment in the RCT but did not meet the inclusion criteria. The second dataset was from 144 enrolled study participants (Group E) which had been obtained during the prior RCT (Clinical Trials.gov, TRN: NCT04814459 on 24/03/2021). All available documents from the prior RCT participants were included in this retrospective analysis. In other words, no additional inclusion/exclusion criteria were imposed so no data were excluded.

Methodology

Individuals from both Group S and Group E were referred from one of the following six senior community centers located in Michigan, United States: Auburn Hills (AH), Novi (N), Saline (S), St. Clair Shores (SCS), Pittsfield (P), and Van Buren County (VB). Unique identifiers were assigned to documents corresponding to Group S participants. The records of Group E participants had been de-identified as a component of the original RCT and were equally distributed with 24 records from each of the six locations.

Four investigators, who were not key personnel during the original RCT data collection, reviewed all Group S screening and Group E initial evaluation documents. Demographic data were extracted, when available, which included participant age, self-identified gender, and referring community center. Additionally, the rationale for study inclusion and exclusion including outcomes of cognition screening and health screening to assure readiness and safety to engage in exercise were retrospectively reviewed. Key features including written comments extracted from each document, the details of the cognition, and readiness for exercise participation tools are described in the following sections.

Cognition screening

The MiniCog was utilized as an initial screening tool to determine cognitive safety to participate in the RCT [12] as it has strong psychometric properties when screening for Alzheimer’s dementia [13]. Furthermore, the MiniCog has high sensitivity and specificity to detect cognitive impairment [15]. The screening asks individuals to recall three non-matching words and to draw an analog clock with a predetermined display time. The test takes approximately three minutes to administer and is deemed appropriate for use in primary care settings. A cutoff score of 3 has been clinically validated; therefore, a score of 3 or less was coded as “not passing” the MiniCog when extracting data for the purposes of this study [16].

If a potential RCT participant did not meet the criterion cut point of passing on the MiniCog, the TMT was administered as a secondary screening tool. The TMT has evidence of good sensitivity to executive functioning, especially cognitive flexibility as well as good inter-rater reliability [17]. While the Trail Making Test (Part A) is a less challenging assessment, Part B emphasizes higher executive cognitive function and places a higher demand on visual search capacity. The TMT is a timed test, including time to correct mistakes, which requires the individual to draw lines sequentially between numbers and letters (e.g., 1-A-2-B-3-C…). The time to complete the test is recorded, with a score of greater than 273 seconds to complete the TMT assessment deemed “not passing” as cognitive impairment is likely [18]. For the purposes of the RCT, if the individual did not pass the cognitive assessments, they were not enrolled, and therefore were allocated into Group S in the current retrospective study.

Pre-participation health screening

**Figure 1:**
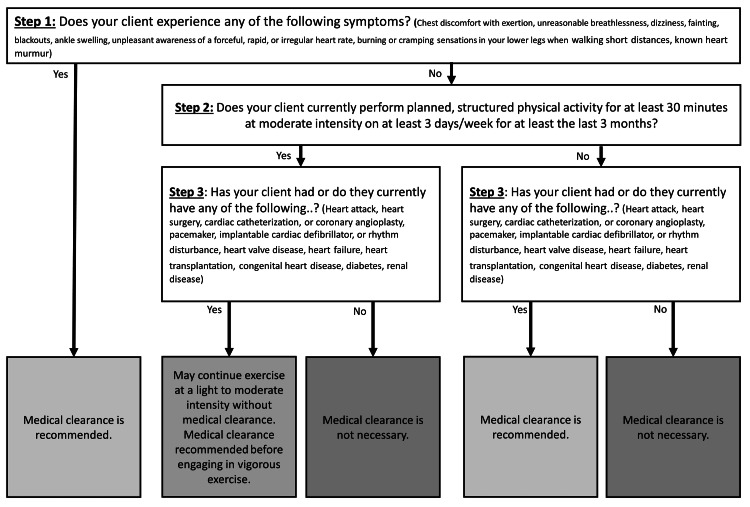
American College of Sports Medicine pre-participation screening algorithm. Modified from Riebe D, Franklin BA, Thompson PD, et al. Updating ACSM’s recommendations for exercise pre-participation health screening. Med Sci Sports Exerc. 2015;47:2473-9. doi:10.1249/MSS.0000000000000664 [9].

The ACSM Exercise Pre-participation Health Screening tool was utilized to identify individuals at an elevated risk for exercise-related sudden cardiac death and/or acute myocardial infarction [9]. This tool was developed with the intent of determining risk prior to initiating exercise. When used by PTs initiating exercise programming in the absence of a physician referral, it provides a mechanism of pre-participation exercise screening without a required face-to-face visit and cost burden to a patient to see the physician or mid-level providers. The tool is used to screen key cardiac risk indicators, including current physical activity levels, active signs or symptoms, and/or known cardiovascular, metabolic, or renal disease within the context of the planned exercise intensity. The tool allows a healthcare provider to identify the risk associated with initiating exercise among individuals with cardiovascular or pulmonary disease or those at risk for sudden cardiac death [19]. The tool has high sensitivity but low specificity, suggesting the survey is unable to identify those with no contraindication to begin exercise; however, it can identify those with a contraindication to begin exercise [19]. If an individual was deemed to be at an elevated risk for an adverse medical event using the tool, further examination and medical clearance prior to initiating an exercise program were recommended as per the protocol during the RCT from which the data in this study were obtained [12]. Figure 1 provides an algorithm detailing the key steps to using this screening tool.

Data analysis

Descriptive statistics were generated to analyze the central tendencies of the demographic and outcome variables. A chi-square test analyzed the differences in cognition-focused outcome variables by age category. Statistical analysis was performed using SAS v.9.4 (SAS Institute, Cary, NC, USA) software for Windows with significance determined at p-values <0.05.

## Results

A total of 214 records were reviewed including 70 from Group S and 144 from Group E.

Group S demographics

Group S records, individuals who were excluded prior to HOP-UP-PT program enrollment, were referred from the six different senior community centers located in the corresponding municipalities within Michigan: AH = 27.1% (n = 19), N = 11.4% (n = 8), S = 4.3% (n = 3), SCS = 7.1% (n = 5), P = 11.4% (n = 8), and VB = 30.0% (n = 21) with 8.6% (n = 6) of the records from an unreported location. Of Group S records, 67.1% (n = 47) identified as female, 8.6% (n = 6) as male, and 24.3% (n = 17) did not specify gender. While 81.4% (n = 57) of Group S referrals were from individuals who met the inclusion criteria of being greater than or equal to 65 years of age or older, 4.3% (n = 3) were excluded as they were less than 65 years old, and age was not available in 14.3% (n = 10) of the referral documents.

Group S cognition screening and assessment

**Figure 2:**
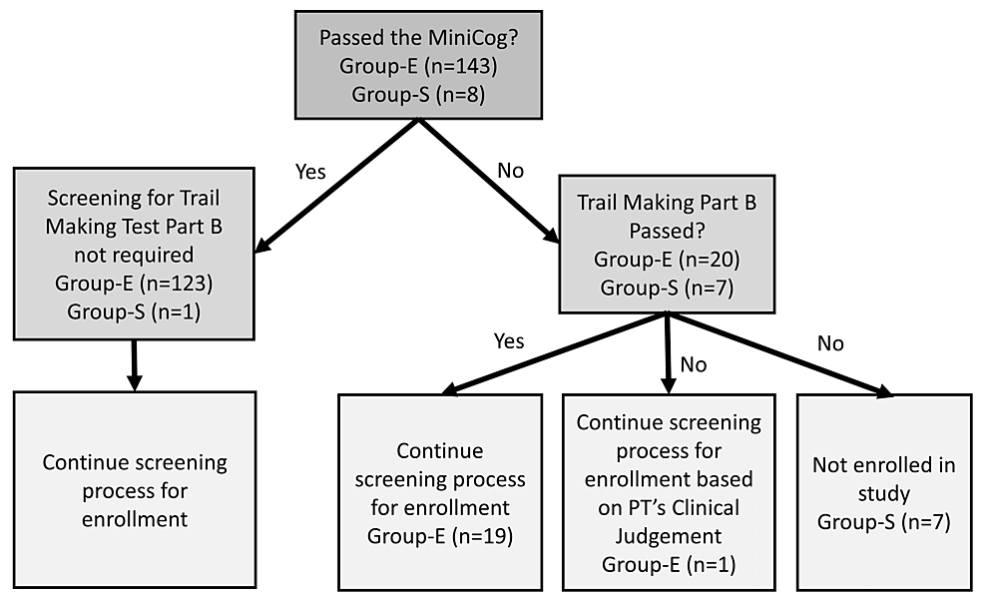
MiniCog and Trail Making Test-Part B results for Groups S and E. PT = physical therapist

Records from Group S indicated that three individuals responded “yes” to having a diagnosis of Alzheimer’s or confusion on the referral sheet. Additionally, the MiniCog administered during an in-person enrollment screening visit of eight individuals did not meet the criterion passing score. After administering the TMT, seven individuals were not enrolled as they did not pass either cognitive screen. These records are, therefore, included in the Group S analysis. The one individual who did pass the TMT was excluded due to a recent medical event. Figure 2 depicts the results of the MiniCog and TMT by step for both Group S and Group E.

Group S additional exclusion outcomes

Following initial evaluation and inclusion/exclusion screening, three (4.3%) records indicated that the individual required traditional, episodic, impairment-focused physical therapy with comments including “wanting to start physical therapy now” and “acute right shoulder injury” included in the documentation as the supportive rationale for exclusion. Data extraction identified that 2.9% (n = 2) of the records were from individuals deemed not medically appropriate for a prevention-focused therapy intervention, with recent knee surgery and acute shoulder injury included as the supportive rationale. Additionally, Table 1 describes the percentage and frequency of non-enrollment and the rationale for exclusion.

**Table 1:** Percentage, frequency, and rationale for Group S enrollment exclusion.

Rationale for exclusion	% (n)
Age <65 years old	4.3 (3)
Diagnosis of Alzheimer’s or confusion	4.3 (3)
Individual did not pass MiniCog or Trail Making Test Part B	10.0 (7)
Hospitalization in the past two months	4.3 (3)
Participation in physical therapy in the past two months	12.9 (9)
Individual had been told that exercise was unsafe for them	2.9 (2)
Individual declined participation	45.7 (32)
Inability to contact individual	15.7 (11)

Group E demographics

Group E participants were referred equally from each of the six previously identified communities (16.7%, n = 24). Of those enrolled in the study, 75.7% (n = 109) identified as female, 23.6% (n = 34) as male, and 0.7% (n = 1) did not list a gender. The age categories represented in the Group E documents were as follows: 40.9% (n = 59) were 65-74 years old, 43.8% (n = 63) were 75-84 years old, and 15.3% (n = 22) were greater than or equal to 85 years old.

Group E inclusion outcomes identified during the initial evaluation

No individuals were identified as needing standard-of-care physical therapy or deemed to have medical concerns that would exclude them from the preventative exercise programming of the RCT by their evaluating PT during the initial evaluation, therefore all 144 met the inclusion criteria for participation.

Group E cognition screening and assessment

Records from Group E participants identified that 143 of the enrolled participants were administered the MiniCog with one individual unable to perform due to self-reported vision impairment (e.g., legally blind). As depicted in Figure 2, 86% (n = 123) of records indicated that these individuals passed the MiniCog. Of the 20 who did not pass, 19 went on to pass the TMT Part B and were able to fully participate in the program. One individual did not pass either the MiniCog or the TMT but was inadvertently enrolled in the study. Notation from the evaluating PT indicated “a distracting/noisy environment” as the rationale for the older adult not achieving a passing score on both cognitive tests.

ACSM pre-participation screening

**Figure 3:**
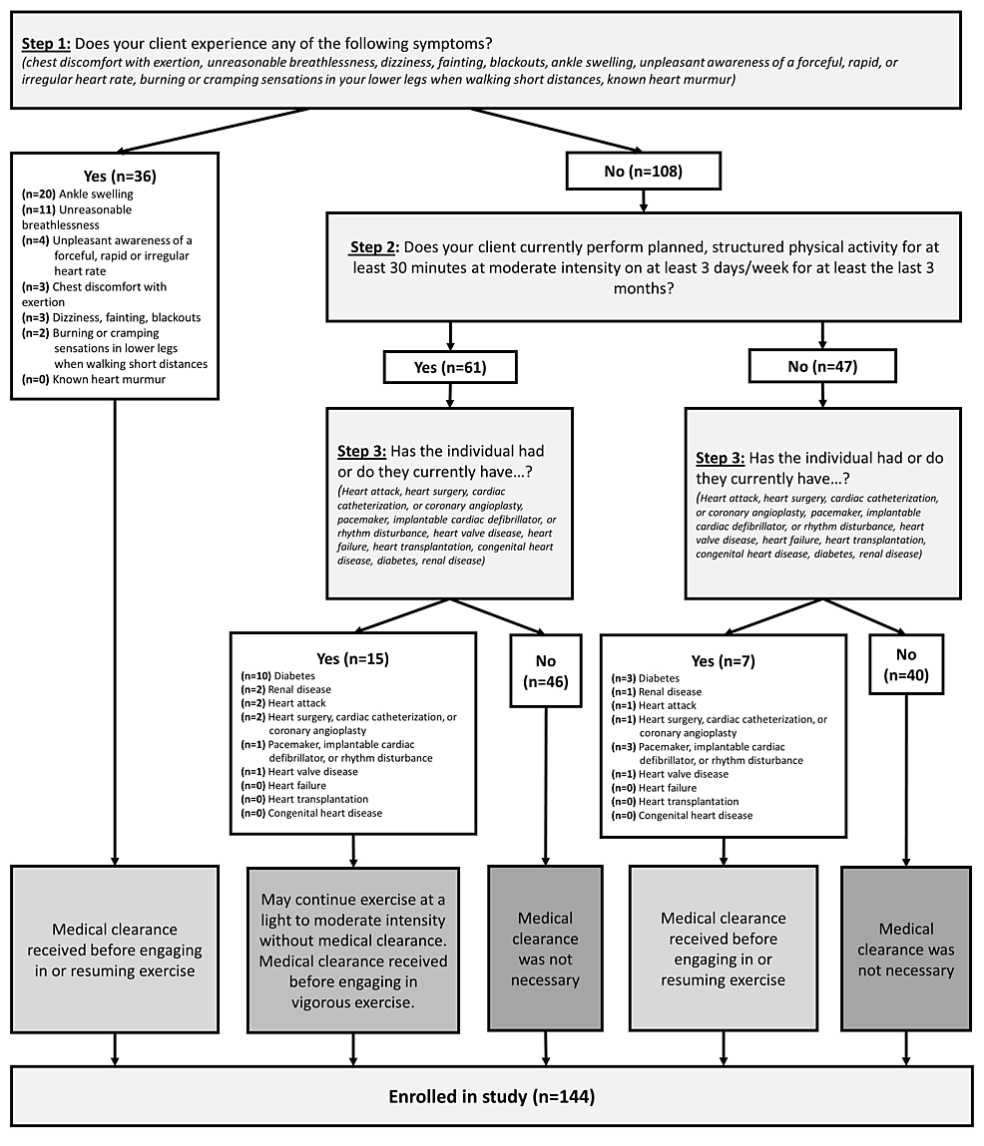
American College of Sports Medicine Pre-participation Screening outcomes.

Table 2 and Figure 3 detail the outcomes of the ACSM Pre-participation Screening tool data from Group E. Action by the PT to obtain medical clearance prior to participation in the program was required and received from a physician or physician extender for 40.3% (n = 58) of participants.

**Table 2:** American College of Sports Medicine Pre-Participation Screening results

Action needed prior to enrollment	% (n) of individuals meeting the criterion
Medical clearance not necessary	59.7 (86)
Exercise at moderate intensity permitted without medical clearance. Medical clearance for vigorous intensity	10.4 (15)
Medical clearance from physician required	29.9 (43)
Total participants requiring some level of medical clearance	40.3 (58)

Differences in cognition outcomes by age category

Table 3 depicts cognition pass rates for each measure among Group E participants by age category. A significant difference was identified among Group E participants’ MiniCog passing rate by age (p = 0.02). In other words, MiniCog fail rates increased with advancing age. However, this was not observed with the TMT.

**Table 3:** Group E differences in cognition-focused outcome variables by age.

Age (years)	% of Group E	% passed MiniCog	% passed Trail Making Test Part B
65–74	41.0	91.5	100
75–84	43.8	87.3	90
85+	15.3	66.7	100
P-value		0.02	0.59

Group E safety outcomes

Importantly, no individuals in Group E were identified as having any adverse effects or significant medical events as a result of participating in the seven-month prevention-focused exercise program.

## Discussion

The purpose of this study was to determine if the use of evidence-based cognitive and cardiovascular screening prior to initiating a prevention-focused exercise program that utilizes a PT direct consumer access referral model is safe. Direct access to PT services provides an opportunity for cost-effective care with significant improvements in functional outcome measures when compared to physician-first access [20]. This study identified that when utilizing high-quality standardized screening tools, PTs were able to identify when a person was not safe for enrollment or required medical clearance prior to safely participating in prevention-focused exercise after being referred from community centers. The rationale for non-enrollment included a therapist’s determination that the person needed traditional tertiary PT interventions or having a recent hospitalization which may present less predictable patterns in exercise tolerance. Additionally, evidence-based tools assisted PTs in identifying cognition or cardiovascular risks and prompted the need for physician clearance. Therefore, these tools appear to be valuable screening adjuncts to identifying potential safety risks of older adults prior to engagement in PT-led prevention-focused healthcare services.

This study’s finding of 40.3% of individuals requiring physician clearance prior to initiating exercise is lower than that reported by Whitfield et al. [19]. Specifically, prior evidence has identified 54.2% of all persons greater than 40 years, 66.2% of those 65-69 years, and 74.6% of individuals over 70 years required clearance before initiating any level of exercise [19]. However, when comparing the need for clearance for vigorous exercise specifically, the 10.4% findings of the current study exceed the 1.9% finding for all individuals greater than 40 years old previously reported [19]. This discrepancy continues when contrasting the 65-69-year-old category (3%) and the 70 and older categories (1.8%) of the Whitfield study to the findings of the current study [19]. While the rationale for these differences requires further examination, it is plausible that the “at-risk” referral criteria employed in this study contributed to this variation. However, what is known is that the ACSM Pre-participation Screening tool was useful to PTs in identifying those who should be medically cleared prior to participating in either moderate or vigorous exercise and can be a useful tool to incorporate into direct access referrals to PTs. In addition to this tool, measures of exercise intensity including heart rate, Borg rating of perceived exertion, and the talk test should be utilized throughout the course of care with person-centered education on self-monitoring to optimize the safety of exercise prescription and intensity [6,21].

The significant relationship between an individual failing the MiniCog and advancing age was expected. However, this finding further emphasizes the importance of screening older adults for cognitive impairments as these deficits may limit an individual’s ability to age safely in place or participate safely in unsupervised home exercise programming. While this study used two valid and reliable cognitive screening tools to assure safe participation, one participant inadvertently remained in the study even after not passing either cognitive screen. While the individual subsequently safely participated in the entirety of the program without incident, this finding may suggest future use of a cognitive tool that includes the screening of contextual factors such as the presence of distractions in the surrounding environment during testing, an overall understanding of safety considerations of the prescribed exercise program, caregiver support, and current medical status. However, further research to support this isolated finding is warranted.

PTs functioning as direct access healthcare providers are capable and well-positioned to screen for potential risks related to exercise safety. PTs have the necessary skills to identify medical risk factors and then incorporate body structure impairments, functional limitations, and participation restrictions into a safe exercise prescription throughout an episode of care. This should also include considerations for independent exercise programming beyond direct intervention [8]. A PT’s ability to accurately identify individuals who are unfit for participation in a preventative PT program is crucial to maintaining the integrity and safety of the exercise programming, as well as maintaining the trust of potential participants and the medical community. Moreover, this study found that PTs accurately and appropriately consult with physicians and mid-level providers when concerns are identified to confirm the safe initiation of exercise.

Prevention-focused programming using tools with strong psychometric properties for identifying risk provides a framework to support PTs as direct access providers. While the screening tools used in the current study (ACSM Pre-participation Screening, MIniCog, TMT) were utilized as a component of the original RCT inclusion/exclusion criteria, these tools are routinely used by PTs in clinical practice and can therefore translate to use in direct access PT practice patterns. Support for direct access PT services has been described in a prior systematic review which identified direct access physical therapy as producing desirable outcomes, including being capable of achieving more goals, increasing patient satisfaction, reporting less pain at discharge, and requiring less adjunctive testing when compared to individuals who received a physician referral to physical therapy [22]. Additionally, without PT screening and guidance related to safe exercise programming, an older adult who independently began exercise without medical consultation may have plausibly initiated exercise in an unsafe manner, thereby increasing the risk of a medical or fall event. The finding of the current study supports PT direct access referral mechanisms from the community as an opportunity to bridge the gap between public health and medical care delivery options for older adults and increases access to safe exercise participation for at-risk older adults.

Study limitations

Limitations of this study include that the data analyzed were from one RCT with a specific geographic reach in the lower peninsula of Michigan and with participants who were predominantly female. This limits the generalizability to broader populations. Additionally, referral to PTs was from community senior centers, and, therefore, it is plausible that outcomes may differ if using a different direct access referral source (e.g., older adult self-referral). Finally, the retrospective nature of this study limited the investigators’ ability to explore clinician rationale and decision-making beyond the documented findings.

Future research

Future research with an expanded geographic reach, intention toward prospectively garnering data on safety-related outcome measures, and assessment of the efficacy of other safety screening tools is warranted. Investigation into preferred communication tools for this population and adjunctive opportunities for technology education would also be of benefit. Additionally, further investigations should be designed with the intent to garner insight into various reasons that participants declined to participate in the study.

## Conclusions

PTs must incorporate cardiovascular and cognitive screenings with strong psychometric properties to assure safety for older adults prior to engagement in PT-led prevention programs. Specifically, the ACSM Pre-participation Screening, MiniCog, and TMT tools are valuable screening adjuncts to identifying potential safety risks of older adults prior to engagement in PT-led prevention-focused healthcare services as the RCT resulted in no adverse effects for any of the 144 older adult participants. Additionally, direct consumer access referrals to PTs from community senior centers offer a safe option for older adults to participate in an individualized exercise that may reduce falls and health-related risks. Direct access to PT is available throughout the United States. However, reimbursement considerations need further examination to assure the skill of the PT is fairly compensated for programs, such as HOP-UP-PT, that can reduce access burdens and improve healthcare value.
